# Mapping current and future habitat suitability of *Azolla* spp., a biofertilizer for small-scale rice farming in Africa

**DOI:** 10.1371/journal.pone.0291009

**Published:** 2023-12-18

**Authors:** Xorla S. Ocloo, Gonzalo M. Vazquez-Prokopec, David J. Civitello

**Affiliations:** 1 Department of African and Black Diaspora Studies, DePaul University, Chicago, IL, United States of America; 2 Department of Environmental Science and Studies, DePaul University, Chicago, IL, United States of America; 3 Department of Environmental Sciences, Emory University, Atlanta, GA, United States of America; 4 Department of Biology, Emory University, Atlanta, GA, United States of America; ICAR-Indian Institute of Soil Science, INDIA

## Abstract

How do we feed the expanding human population without excessive resource depletion or environmental degradation? Recycling and recapturing nutrients could alleviate these challenges, especially if these strategies are robust to climate change. Co-cultivating rice with *Azolla spp*. in Asia has demonstrated high yields with reduced fertilizer inputs because *Azolla* fixes atmospheric nitrogen, limits nitrogen volatilization, recaptures and releases other nutrients, and suppresses weeds. While *Azolla* is distributed in Africa, this approach has not been widely implemented in African rice-farming. Characterizing the suitability of *Azolla* is critical in evaluating the potential for *Azolla*-rice in Africa. To do so, we synthesized 189 field and greenhouse studies from around the world that quantified temperature-dependent growth of *A*. *pinnata* and *A*. *filiculoides* and developed present and future climate suitability maps at the continental scale using mean temperatures under two Representative Concentration Pathways. Currently, most of Africa is suitable for *Azolla* with slight differences in regional suitability for each species. We project little change in the continent-wide suitability for both species, but anticipate a regional decline, particularly for *A*. *filiculoides* in the Sahel. Collaborating with farmers to validate these projections, evaluate the costs and benefits of *Azolla*-rice, and facilitate adoption of viable strategies can facilitate equitable food systems that also empower African farmers.

## Introduction

The world population continues to grow rapidly, posing threats to global and local food security [1]. To meet this demand, farmers are pressured to use intensive farming practices such as pesticides, herbicides, and chemical fertilizers to improve crop yields [2, 3]. Specifically, the use of chemical nitrogen fertilizers can endanger soil health by reducing the abundance and activity of beneficial microbes [4], contribute to greenhouse gas emissions, and pollute the ground and surface waters which can have negative effects on aquatic ecosystems, human health, and the economy [5, 6]. Moreover, most farmers in developing countries operate under a small-scale agriculture model and have difficulties accessing resources, such as mineral fertilizers, capital, information and technology, making it difficult for them to enter and compete in world markets [7]. Thus, it is necessary to examine efficient use and alternative sources of nitrogen to meet crop demand with strategies that are equitable, sustainable, and involve social-ecological systems thinking to address complex sustainability challenges.

Rice is a staple crop in West Africa and Madagascar, and is increasingly becoming an important food source throughout Africa [8]. However, rice production poses many challenges for African farmers [8], particularly those in the Sahel region where rice production is concentrated [9]. For example, lack of nutrient-rich soils and the availability and affordability of nitrogen fertilizers, a key input for high yielding rice varieties, make it difficult for African farmers to meet their total nitrogen demand required to produce a successful yield [10]. Nutrient recapture/recycle systems, in which a biological agent captures or fixes nutrients from the environment into usable product [11], are a potential solution. One notable example of this process occurs within legumes and the soil bacteria, Rhizobia [12]. Rhizobia fix nitrogen after establishing inside the root nodules of legumes [12, 13] and improve soil fertility [13]. Recently, the aquatic fern, *Azolla* spp., has gained increasing interest as an effective tool for nutrient recapture/recycle in sustainable agricultural development. The most distinguishing characteristic of *Azolla* is its symbiotic relationship with the nitrogen-fixing cyanobacterium, *Anabaena azollae* [14]. The cyanobacterium is capable of fixing atmospheric nitrogen to ammonium in excess of the plant’s needs [14, 15]. The *Azolla*-*anabaena* pair can fix ∼30-100kg N/ha/month under optimal conditions [15, 16], an estimate ∼6-fold as large as legumes, which fix ∼5-15kg N/ha/month [17]. Fresh *Azolla* growth increases nitrogen concentration in water by 3% [18] and when *Azolla* decomposes, it releases nutrients (e.g., nitrogen, phosphorus, potassium, etc.) into the water [19]. Though *Azolla* can lead to eutrophication when mismanaged in water bodies [20], it can serve tremendous benefits when grown with irrigated agricultural crops [15]. When co-cultivated with crops, *Azolla* can also suppress common weeds [14]. This occurs once *Azolla* forms a thick mat, starving weed seedlings of sunlight and prohibiting their emergence [14].

**Fig 1:**
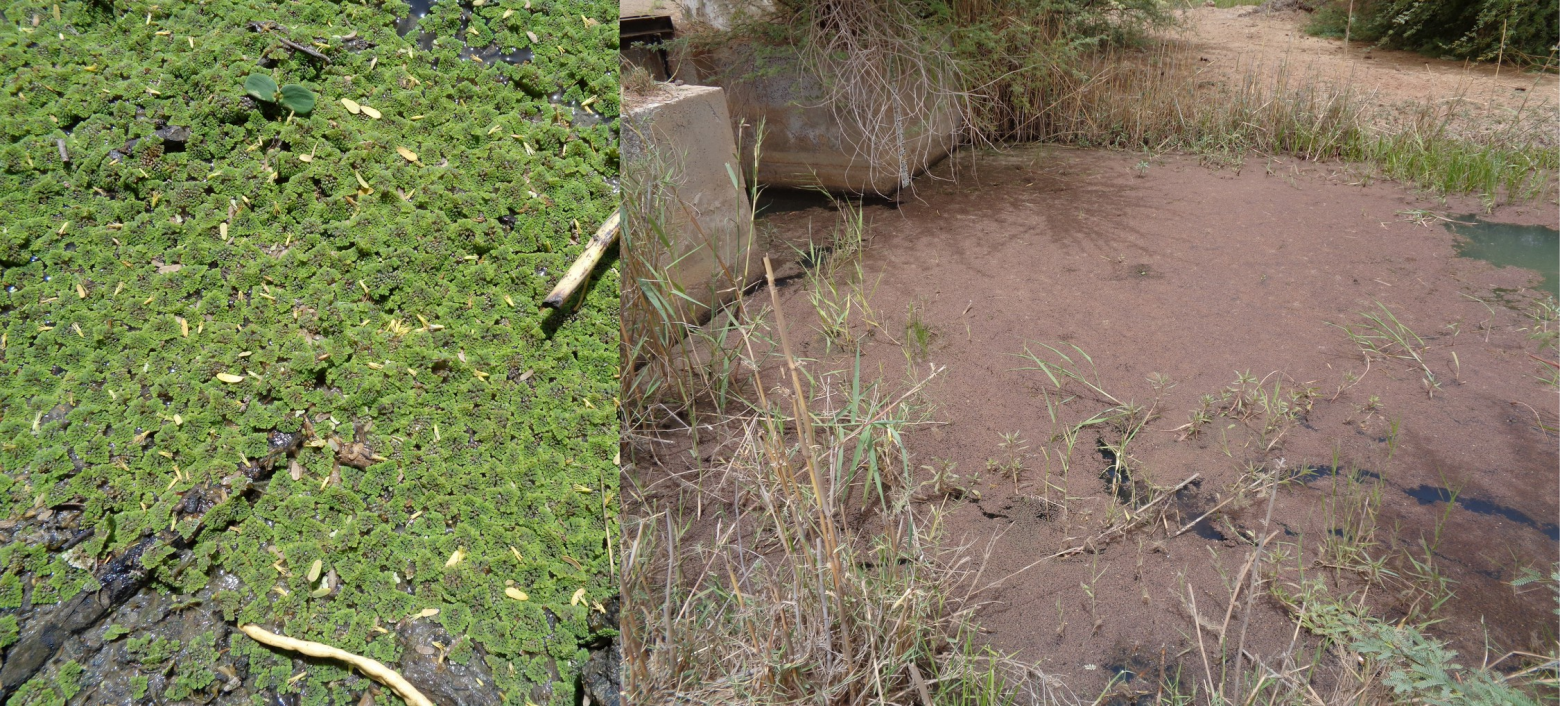
Healthy green *Azolla* spp. (on the left) and decomposing red *Azolla* spp. (on the right) induced by high temperatures found in a canal in Saint-Louis region of Senegal. Photo taken by author XSO in 2019.

Historically, farmers in Asia have already integrated the aquatic fern into low-input sustainable farming systems to fertilize their rice paddies [21, 22]. In China, *Azolla* increased rice growth and also mediated CH_4_ transport by evaporation from flooded rice soil into the atmosphere [23]. In India, *Azolla* increased rice height and tiller number [24]. A small-scale experiment in the United States also demonstrated the effectiveness of *Azolla* as a biofertilizer on rice [25]. For example, *A*. *filiculoides* increased rice yields by 112%, 23%, 216% when incorporated as a basal manure, grown alongside rice, and applied using both techniques, respectively [26]. Despite these advantages and the global distribution of the *Azolla* genus, it has not been co-opted widely outside of Asia and most of the research exploring *Azolla* adoption has been done before the 2000s. There is a lack of awareness of the potential of *Azolla*-rice farming in Africa that could be addressed with further study and science communication. Based on participant observations and interviews with Sahel rice farmers in the Saint-Louis River Valley in northern Senegal, there is a renewed interest for *Azolla* due to increasing demand for affordable sources of nitrogen. Senegalese farmers revealed that they would consider using *Azolla* if there were environmental and economic benefits to rice production, their main priority and goal (unpublished interview data, XSO). Therefore, a critical gap is to evaluate if *Azolla* could provide comparable benefits for African rice farming as it does elsewhere despite differences in regional and local environmental factors in the face of complex futures due to climate change. For example, *Azolla* is known to be highly sensitive to temperature, exhibiting slow growth at low temperature and die-offs in summer months or in response to heat waves [27–29] (Fig 1). Therefore, predicting the performance of *Azolla* under current and future temperatures in rice-farming regions in Africa is a critical step towards equitable implementation of this practice.

In a broader context, mapping *Azolla* suitability is a preliminary step to understanding whether *Azolla*-rice farming can be a valuable agricultural practice in Africa and can be leveraged by the social-ecological systems (SES) framework. The SES framework was developed as a diagnostic tool for assessing sustainability, and recognizes the complex and interdependent relationship between biophysical and social systems [30, 31]. A key strength of the framework is understanding many dimensions of system functioning and seeks to understand all aspects related to the development, implementation, and transformation towards normative societal goals that are also sustainable [32, 33]. In order to understand the feasibility of *Azolla*-rice system as a nutrient/recapture system and promising biofertilizer in Africa, it is important to first address whether Africa’s climate is suitable for *Azolla* using two of the variables of the SES framework that focus on the resource unit (RU, *Azolla* in this application) (1) growth and replacement rate and (2) spatial and temporal distribution. Our aims are to integrate published estimates of *Azolla* growth across thermal gradients to establish thermal performance curves for two well studied species in the genus. Next, we will synthesize these performance curves with current and future climate scenarios (RCP 4.5 and 8.5, intermediate and pessimistic emission scenarios, respectively) to map current and future suitability for *Azolla* in Africa. We predicted that *Azolla* suitability in the Sahel region will be most impacted by climate change because monthly mean temperatures in that region are more frequently at or above temperatures typically cited as optimal for *Azolla*. Understanding the temperature-dependent growth rate and the temporal and spatial suitability, as well as adaptability to climate change, will be important in accurately communicating risks and benefits to African farmers. Additionally, these mapping efforts will help establish equitable implementation plans in places and times when *Azolla*-rice has the greatest potential to succeed.

## Methods

### Mapping habitat suitability

We assessed habitat suitability of *Azolla* for continental Africa using thermal performance curves (TPCs), annual temperature changes, and relative growth rate. To map the spatial and temporal suitability of *Azolla* spp. using the relative growth rate parameter, we compiled empirical data from the literature that examined the effects of temperature on the growth rates of various *Azolla* species using the scholarly databases, Web of Science and Google. We used the key words, “*Azolla*”, “*pinnata*”, “*filiculoides*”, “*nilotica*”, “*microphylla*”, “*mexicana*”, “*caroliniana*”, “temperature”, “relative growth rate”, and “biomass”, to search for all relevant empirical data. To satisfy our inclusion criteria, a study had to report the growth rate or biomass production of at least one species of *Azolla* in at least two temperature schemes. For each included study, we recorded the species, strain, temperature, initial and final time, relative growth rate, standard deviation, and other environmental conditions tested with temperature when applicable (i.e., added phosphorus, elevated CO_2_, changes in pH, various light intensities) (data in S1 Data). When relative growth rate (RGR) was not given, we manually calculated RGR if the initial mass, final mass, and time was provided using the definition of RGR:

$$RGR=\frac{ln\left(Mass_{2}-Mass{\;}_{1}\right)}{\left(Time{\;}_{2}-Time{\;}_{1}\right)}$$ (1)

Where: RGR = relative growth rate from initial time to final time expressed as g/g per day

Mass_2_ = mass in grams at the end of growth period

Mass_1_ = mass in grams at the beginning of growth period

Time_2_ –Time_1_ = time interval of the growth period expressed as days.

When doubling time (DT) was only given, we used the following equation based on the definition of doubling time equation to calculate RGR:

$$DT=\frac{ln(2)}{RGR}$$ (2)

Where: DT = time it takes for a population to double in size expressed in days

RGR = relative growth rate from initial time to final time expressed as g/g per day

When data were not recorded in tables and only in graphs, we used Plot digitizer (http://plotdigitizer.sourceforge.net), an open source software to manually extract estimated information (i.e., temperature and biomass, etc.). In total, we had 282 data points between six species of *Azolla*: *A*. *caroliniana*, *A*. *filiculoides*, *A*. *mexicana*, *A*. *microphylla*, *A*. *nilotica*, and *A*. *pinnata*. The final data consisted of a total of 189 records from *A*. *pinnata* and *A*. *filiculoides* (data in S1 Data). We only used data from *A*. *pinnata* because it is native to Africa and *A*. *filiculoides* because it is globally distributed and used in rice cultivation.

The focus of our literature review was to estimate how temperature changes caused relative differences in *Azolla* RGR by fitting TPCs for *A*. *pinnata* and *A*. *filiculoides* using maximum likelihood estimation strategies, using a similar approach to ongoing efforts to project changes in the distribution of vector-borne diseases, pests, invasive species, and other applications [34, 35]. A diversity of equations to represent TPCs exist. We chose to fit the Room model [36] because it is capable of representing unimodal, asymmetric curves, which are extremely common. It has additional advantage over several other TPC equations in that it has only four parameters which have direct interpretation, e.g., a parameter for the optimal temperature and another for peak performance (maximum growth rate). Lastly, growth rate is defined at all temperatures, an important benefit that is not true for all TPC equations. While the Room model does not have a mechanistic or biochemical interpretation, this drawback is not critical, because we are mapping distribution based on performance, not assessing mechanism:

$$P(T)=\left\{\begin{array}{l} P_{max}exp\left[-a{\left(T-T_{opt}\right)}^{2},\;T\;\leq \;Topt\right]\\ P_{max\;}exp\left[-b{\left(T-T_{opt}\right)}^{2},\;T\;\geq \;Topt\right] \end{array}\right\}$$ (3)

Where: *a* > 0 and *b* > 0 are in units of°C ^−1/2^

P_max_ = peak height of the curve

T_opt_ = Optimal temperature

*a* = slope of the rise of curve at temperatures below the optimal temperature

*b* = slope of fall of curve at temperatures above the optimal temperature

We then used the datasets for *A*. *pinnata* and *A*. *filiculoides* to estimate the parameters of the Room model for each species using maximum likelihood estimation [37]. We assumed a normal error distribution for the model. We also acknowledged that there could be pronounced differences in the maximum growth rate of *Azolla* from study to study. These differences could be attributable to variation in strains or genotypes, environmental conditions other than temperature, and the skill or decisions of experimenters. To account for this variation, we incorporated a random effect of study on the peak height parameter, *a*, using an integrated likelihood approach [38]. We conducted our model fitting analysis using the R package bbmle [39].

### Mapping current and projected suitability for *Azolla*

We mapped the current and projected suitability of *Azolla* by inputting selected climatic data into the thermal performance curves that we parameterized for *A*. *pinnata* and *A*. *filiculoides*. We used current global annual temperatures (bioclimatic variable 1) for the climatic period of 1970–2000 at the spatial resolution of 5 minutes (∼18.5 km at the equator) taken from the WorldClim Database [40]. For future climate predictions, we also used the annual temperatures (bioclimatic variable 1) from downscaled IPPC5 Coupled Model Intercomparison Project Phase 5 (CMIP5) [41] by the World Climate Research Programme, provided by WorldClim for 2050 (averaged for 2041–2060). The data available used the global circulation model, GISS-E2-R, and included four Representative concentration pathways (RCPs): RCP 2.6, 4.5, 6.0, and 8.5. For this study we selected an intermediate projection, RCP 4.5, and a pessimistic scenario, RCP 8.5, to model both possibilities [42]. For both sets of current and future data, we clipped the region to the African continent and computed the relative growth rate (RGR) of each *Azolla* species from the fitted thermal performance curves and the estimated temperature at each pixel for all scenarios. We then generated maps to visualize suitability across Africa.

## Results

### Thermal performance curves

**Fig 2:**
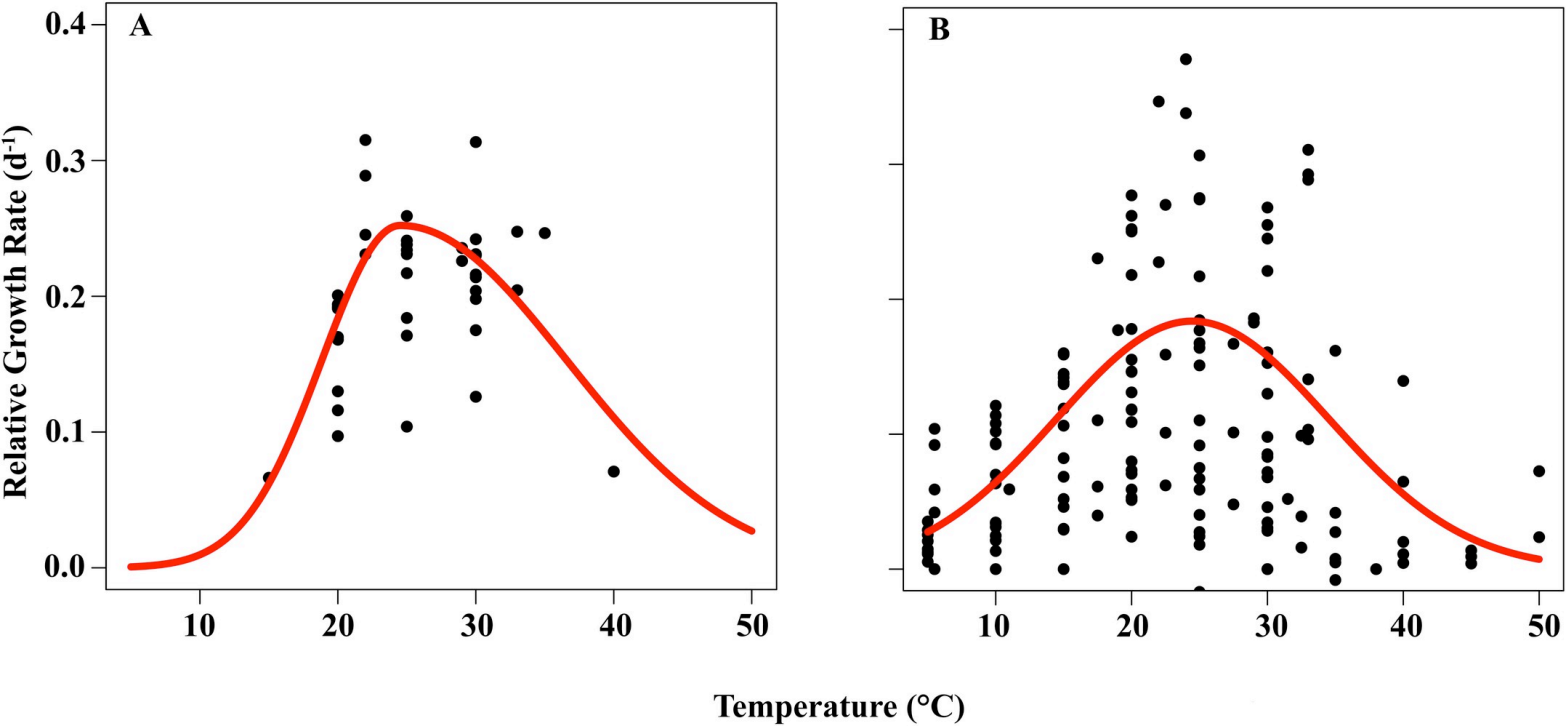
Overall mean thermal performance curves (lines) for (A) *A*. *pinnata* and (B) *A*. *filiculoides* estimated from experiments conducted over temperatures of 5°C—50°C. Each point represents the mean estimated relative growth rate from a single temperature treatment in a primary study. Points represent 40 studies for *A*. *pinnata* and 149 studies for *A*. *filiculoides* showing T_opt_ = 24.5°C for both species, average maximum growth rate, P_max_ = 0.25 d^–1^ for *A*. *pinnata* and P_max_ = 0.18 d^–1^ for *A*. *filiculoides*.

We used thermal performance curves to estimate how the growth rate of *Azolla spp*. depends on temperature. The two species had similar optimum temperature (parameter *Topt* = 24.5°C), but they differed in the shapes of their TPCs (Fig 2). Specifically, *A*. *pinnata* displayed steeper increases in performance below its optimal temperature (parameter *a* = 0.0155 for *A*. *pinnata* vs. 0.005 for *A*. *filiculoides*) and shallower declines in performance above its optimal temperature (Fig 2A) than *A*. *filiculoides* (parameter *b* = 0.00344 for *A*. *pinnata* vs. 0.00495 for *A*. *filiculoides*) (Fig 2B). These parameterizations resulted in curves that are consistent with the generally recognized pattern that *A*. *pinnata* is more heat-tolerant than *A*. *filiculoides* (Fig 2).

### Current and future projections of *Azolla* spp. in Africa

To predict *Azolla* spp. current and future spatial and temporal distribution in Africa, we used the thermal performance curve parameters to estimate the suitability for *Azolla* spp. across Africa. Our current prediction map shows that most of Africa has temperatures suitable for the production of both *Azolla* species (S1 Fig). The average relative growth rate across Africa for *A*. *pinnata* was 0.220 d^-1^ (S1A Fig) and 0.172 d^-1^ for *A*. *filiculoides* (S1B Fig) for current temperatures (Table 1). We found that countries in central Africa have the highest suitability for both *Azolla* spp. compared to countries in the north, east and south of Africa (S1 Fig). We found that *A*. *pinnata* has a higher growth rate than *A*. *filiculoides* in the Sahel region (S1 Fig). *Azolla filiculoides* was better suitable for countries in the north, east, and south of Africa compared to *A*. *pinnata*. Our future prediction map for RCP 4.5 showed that the overall average relative growth rate of *A*. *pinnata* was 0.228 d^-1^ and 0.171 d^-1^ for *A*. *filiculoides* across Africa (Table 1). For the pessimistic scenario (RCP 8.5), the relative growth rate of *A*. *pinnata* was 0.229 d^-1^ and 0.171 d^-1^ for *A*. *filiculoides* (Table 1). Based on future climate predictions, *A*. *pinnata* is expected to perform better under RCP 4.5 (S2A Fig) than RCP 8.5 in the Sahel region (S2C Fig). *A*. *filiculoides* is also expected to have greater suitability under RCP 4.5 (S2B Fig) than RCP 8.5 (S2D Fig).

**Table 1:** Relative growth rate of *Azolla pinnata* and *A*. *filiculoides* based on current mean temperatures and future mean temperatures projected under two future emission scenarios in one time period.

	Relative Growth Rate (d^-1^) Current ∼1970–2000	Relative Growth Rate (d^-1^) Future ∼ 2050	Relative Growth Rate (d^-1^) Future ∼ 2050
		RCP 4.5	RCP 8.5
***Azolla pinnata***	0.220	0.228	0.229
***Azolla filiculoides***	0.172	0.171	0.171

### Visualizing the difference between current and future climate projections of Africa

**Fig 3:**
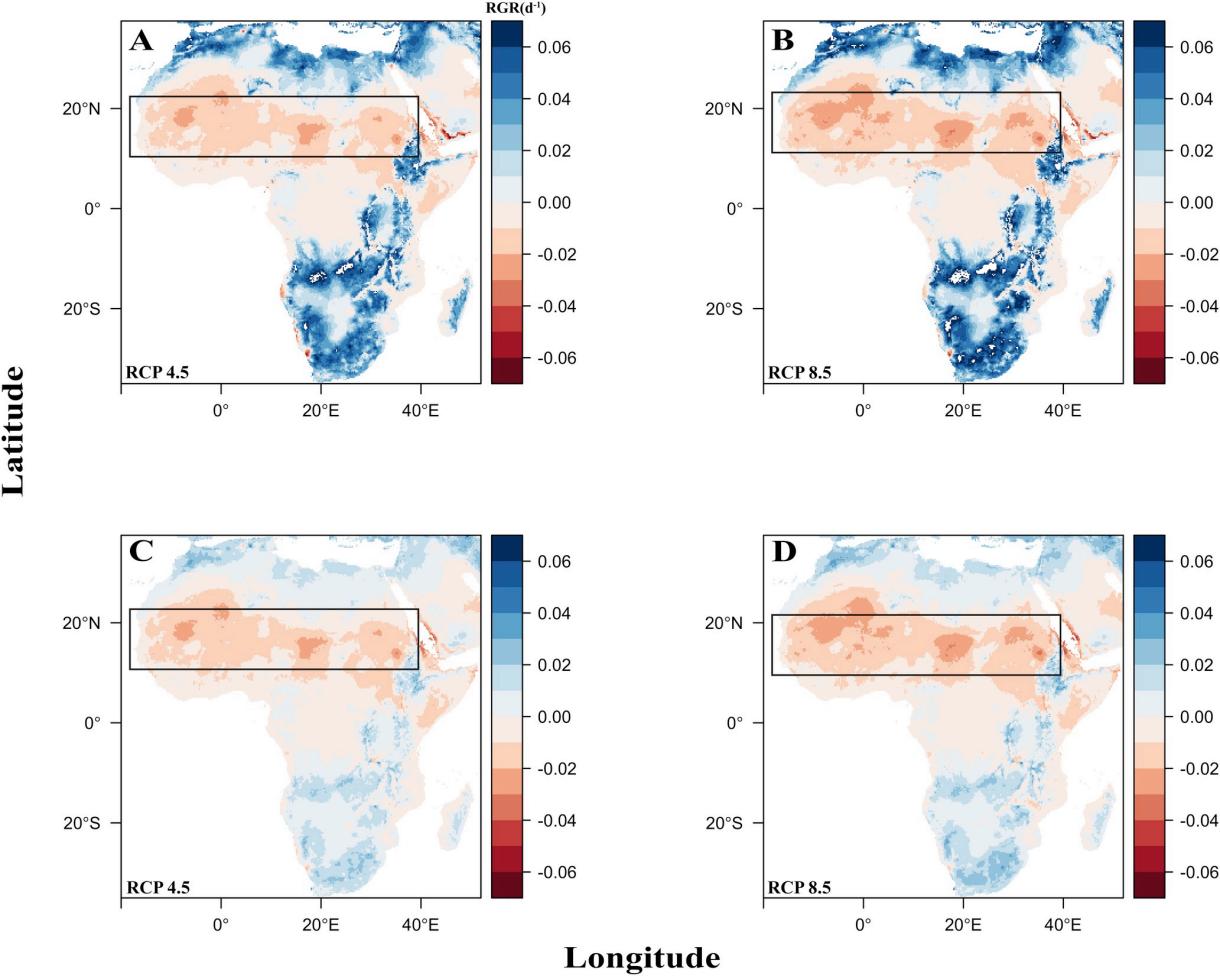
Suitability maps highlighting change in relative growth (RGR) and potential habitats of *A*. *pinnata* (A and B) *and A*. *filiculoides* (C and D) in continental Africa according to two RCPs. Regions expected to decline is shown in red, while regions expected to increase in suitability is highlighted in blue. For both RCP scenarios, the Sahel region (denoted in the black box) is expected to decrease in *Azolla* spp. suitability, whereas north, east, and south Africa is expected to increase in suitability. The suitability of *Azolla* for many countries in central Africa is projected to be relatively unchanged.

We found that the mean difference between the current and future maps of *A*. *pinnata* for RCP 4.5 was 0.0074 d^-1^ and 0.0084 d^-1^ for RCP 8.5 (Fig 3A and 3B) (Table 2). The mean difference between the current and future maps of *A*. *filiculoides* for RCP 4.5 was –0.00066 d^-1^ and –0.00119 d^-1^ for RCP 8.5 (Fig 3C and 3D) (Table 2). Overall, we found a 3.38% and 3.82% increase for RCP 4.5 and 8.5 respectively in relative growth rate for *A*. *pinnata* and a 0.38% and 0.69% decrease for RCP 4.5 and 8.5 respectively in relative growth rate for *A*. *filiculoides* in the year 2050 (Table 2). On average between both *Azolla* species and both RCPs, the model predicted that productivity would increase in north, east, and south Africa. We also found that on average *Azolla* suitability decreased in the Sahel region as expected since growth rate declines at high temperatures and the Sahel region is the hottest region in Africa (Fig 3). This decline was more pronounced with *Azolla filiculoide*s. All future relative growth rate figures for *A*. *pinnata* and *A*. *filiculoides* can be found in S2 Fig.

**Table 2:** Mean difference and percent change in relative growth rate (RGR) for *Azolla pinnata* and *A*. *filiculoides*.

	Mean difference in RGR (d^-1^) Future ∼ 2050 RCP 4.5	Percent change in RGR Future ∼ 2050 RCP 4.5	Mean difference in RGR (d^-1^) Future ∼ 2050 RCP 8.5	Percent change in RGR Future ∼ 2050 RCP 8.5
***Azolla pinnata***	0.0074	+3.38%	0.0084	+3.82%
***Azolla filiculoides***	–0.00066	–0.38%	–0.00119	–0.69%

## Discussion

Mapping tools for agriculture can show areas that are suitable for crops, symbionts, or other important co-cultivating species across space and time. Our synthesis estimated that the optimal temperature for *A*. *pinnata* and *A*. *filiculoides* is 24.5°C, and that *A*. *pinnata* is more tolerant to high temperatures and that *A*. *filiculoides* is more cold-tolerant than *A*. *pinnata*. Given these thermal performance curves, most of Africa is currently suitable for *Azolla*, consistent with its widespread distribution [42]. These results suggest that farmers in rice growing regions in current Africa can potentially adopt *Azolla*-rice farming as a low-cost and low-input alternative biofertilizer to traditional fertilizers depending on which *Azolla* species are locally available. However, we found that *Azolla* suitability in the Sahel region, where rice agriculture is currently highly concentrated, is likely to decrease while other areas in Africa are predicted to increase with *A*. *pinnata*. Moreover, we found that *A*. *filiculoides* might be a better long-term candidate as a biofertilizer for regions in central Africa by the year 2050 (Fig 3C and 3D).

### Comparing performance curves

The finding that *A*. *pinnata* is more heat-tolerant while *A*. *filiculoides* is more cold-tolerant is concordant with narrative reviews of the literature on *Azolla* spp. The optimal temperature for *Azolla* spp. is between 18°C and 28°C [43], although some species have a wide temperature range between –5°C and 35°C [28]. The optimal temperature for *A*. *pinnata* and several other *Azolla* spp. is 30°C [15]. Growth rate begins to decrease above 35°C [28] and fronds begin to die above 45°C and below 5°C [27, 29]. Although *A*. *pinnata* is widely distributed in the tropics, it grows better in cooler seasons [15]. For example, *A*. *pinnata* grew from July to December but was absent from ponds in the hot summer (April to June) in India [44]. In the Philippines, *A*. *pinnata* growth drastically declines in April and May when monthly average temperatures exceed 32°C [45]. In contrast, *A*. *filiculoides* prefers lower temperatures of 25°C than *A*. *pinnata* [15]. *A*. *filiculoides* could withstand temperatures as low as –5°C but was less tolerant than other *Azolla* species to high temperatures [46]. Temperature is known to affect nitrogenase activity important for N-fixation and *Azolla* reproduction. When comparing nitrogenase activity of temperatures ranging from 10°C to 42°C, *A*. *filiculoides* prefers lower temperatures than *A*. *pinnata* [47]. Because both *Azolla* species have wide thermal performance curves, suitability based on growth rate is not very different between current and future climate data. Integrating more data from extremely low and high temperatures for *A*. *pinnata* will help clarify *Azolla* performance in the extremes of the thermal range. Likewise, continuing to evaluate local *Azolla* strains and additional species such as *A*. *nilotica*, *A*. *mexicana*, *A*. *caroliniana*, and *A*. *microphylla*, across temperatures at many sites will help understand which species is most suitable for a given habitat. Although these finding highlight habitat suitability of *Azolla* across several countries, very few studies highlight suitability in African countries. This study highlights places where *Azolla*-rice has the greatest potential to succeed in Africa, representing regions apart from the Sahel region. Since most of Africa is suitable for the growth and development of *Azolla*, examining how *Azolla* effects the stages of development of rice using field studies will be important in understanding if this practice biologically works in different country case studies.

### Future projections for *A*. *pinnata*

The models under both future projections suggest that *A*. *pinnata* will have an overall higher relative growth rate and habitat suitability in 2050. On average across Africa, *A*. *pinnata* is predicted to have a higher relative growth rate with RCP 8.5, a worse-case climate change scenario, than RCP 4.5, an intermediate scenario. However, we project regional changes in suitability for this species. Specifically, distribution will increase in the northern, southern, and eastern regions of Africa, areas that are generally cooler signifying more suitable areas for *Azolla* production (Fig 3A and 3B). Conversely, the future models predict that the countries in the Sahel region will decrease in *Azolla* habitat suitability, especially with RCP 8.5. If these shifts in suitability are large enough, they could jeopardize west African farmers who are currently interested in *Azolla*-rice cultivation. Although, co-cultivating rice and *Azolla* may be feasible now, it may only be a viable strategy in the intermediate term, if climate change decreases *Azolla* habitat suitability in rice growing regions. For example, Senegal has two main rice growing areas: the irrigated Senegal River Valley and the rainfed Casamance regions [48]. Senegal is likely a candidate for *Azolla*-rice farming because *Azolla* grows natively in their water bodies [49], Senegal highly depends on rice imports, and acquiring fertilizer is a major constraint for rice farmers [50]. However, using *Azolla* as a biofertilizer for small-scale farming may not be recommended in 2050 because it may be difficult to grow *A*. *pinnata* in nursery fields or in rice paddies under warmer climate. This anticipated challenge could be overcome through the identification or cultivation of heat-tolerant or otherwise locally-adapted lineages of *Azolla*.

### Future projections for *A*. *filiculoides*

Overall relative growth rate for *A*. *filiculoides* for both climate change scenarios show a decrease in RGR and habitat suitability over time. Similarly, the countries in the Sahel region will be more affected than areas in north, south and east Africa, and this effect is more pronounced in the worst-case scenario, RCP 8.5. One reason why *A*. *filiculoides* is predicted to decrease in RGR compared to *A*. *pinnata* is because it is not as heat-tolerant. The generally reported optimal temperature of *A*. *filiculoides* is 25°C compared to 30°C for other *Azolla* species [15]. *A*. *filiculoides* is the only species found to withstand temperatures as low as –5°C [46]. The effects of climate change will likely disrupt nitrogenase activity important for N-fixation and *Azolla* reproduction [47]. Many countries in central Africa will experience little to no change of relative growth rate of *A*. *filiculoides*.

### Comparing methods to evaluate *Azolla* suitability

Multiple approaches to model global habitat suitability of a species exist, each differing in the data inputs and prediction algorithms. Here, we conducted an extensive literature review to identify studies that reported the productivity of *Azolla* spp. at different temperatures and used it to model the physiological thermal response of *A*. *pinnata* and *A*. *filiculoides* and to estimate the optimal temperature for the relative growth rate of *Azolla*. We then used this prediction to model change in habitat suitability across Africa based on current and future global annual temperatures (bioclimatic variable 1). A study evaluated the potential global distribution of *Azolla filiculoides* using species distribution models and 8 bioclimatic variables under two future climatic scenarios and two time periods, 2050 and 2080 [51]. This study and our study found that habitat distribution for *A*. *filiculoides* is expected to decrease, and that the species might colonize new geographical areas where it is currently not present. Another recent study also used correlative ecological niche models based on presence-only reports of *Azolla* spp. to predict areas in Africa suitable for *A*. *pinnata* and *A*. *filiculoides* identified similar results as ours [51]. They found that under current climate conditions, using 12 Bioclimatic variables and elevation, the potential habitat range was larger than recorded and that temperature was an important climate variable that affected *Azolla* species’ distribution [51]. While we share similar maps that predict that Senegal, Ghana, Togo, Benin, are highly suitable areas currently for *A*. *pinnata* (S1B Fig), our projected maps using only bioclimatic variable 1 provide further insight that Senegal is projected to experience a decrease in habitat suitability in the future based on relative growth rate. Their projections for future habitat suitability for RCP 4.5 and 8.5 predicted that *A*. *pinnata* would have the largest stable habitat followed by *A*. *filiculoides*, likely due to lower heat-tolerance as we also found in our study (Fig 3). They predict a greater loss in habitat suitability for *Azolla nilotica* across the Sahel [51], but we found that *A*. *filiculoides* and *A*. *pinnata* will also experience a loss in habitat suitability in the Sahel region (Fig 3). Additionally, while our study predicts *Azolla* productivity, the other similar study predicts the probability of species occurrence within a given pixel [51]. The general agreement of both approaches suggests that *Azolla*-rice farming can be pursued with confidence in many regions. On the ground results of such trials could also help evaluate and refine these current and predicted distributions.

### Recommendations

The utilization of *Azolla* spp. in rice production would be beneficial to small-scale farmers, especially those who are resource constrained. Our projections suggest a decrease in *Azolla* productivity in the Sahel region of Africa, therefore it could be a priority to identify more heat-tolerant lineages that can withstand future climate temperatures. Additionally, countries in the Sahel region typically have one to two rice growing seasons [52], therefore *Azolla*-rice farming could work better in one season even if it does not work in both [15, 44]. The timing of *Azolla* usage may also be important when considering *Azolla*-farming in the Sahel. For example, if the best time to grow *Azolla* spp. is during the off season (December–May) when temperatures are cooler, it may be a better time to cultivate *Azolla* in large abundances in preparation for the regular rice season, when it is too hot for the survival of *Azolla* spp. In this case, farmers can take advantage of high temperatures by letting the ferns die and decompose in summer, thus releasing nutrients that are important for rice plants. Lastly, Sahel rice farmers can explore different strategies of incorporating *Azolla* into the rice paddies. For example, making *Azolla* compost during seasons when production is at its highest for the usage in later use. Although our projections predict that Sahel rice farmers might eventually find it difficult to exploit the full potential of *Azolla*, smaller scale climatic variation (site-to-site) could still show that *Azolla*-rice farming can work. To understand this, field experiments are needed to evaluate the best strain and farming strategy for a given habitat.

The social-ecological systems framework allows for the integration of data from the natural and social sciences, which allow scientists and practitioners to tests hypotheses regarding the dynamics and functionality of food systems [53]. Previous work has highlighted that biological, institutional, and social factors increases the likelihood of sustainable systems [54, 55]. If *Azolla*-rice farming is climatically suitable and improves rice production through field experimentation, using the social-ecological systems framework should be the way to facilitate broad adoption. To do so, it is recommended that researchers and practitioners involve local stakeholders to first identify variables relevant to the *Azolla*-rice system. For instance, understanding the economic value of *Azolla* in *Azolla*-rice farming as a resource unit can be an important indicator of adoption for farmers. For example, a higher net economic benefit was found when replacing urea with *Azolla* over a 3-year period [16]. Investigating other biological variables defined within SES, such as “Interactions Among Resource units”, may be important when dealing with systems that contain invasive weed species or animals (i.e., fish, ducks, snails). Also, exploring the government and nongovernment organizations involved in *Azolla*-rice farming will help in understanding whether farmers who adopt the practice will also receive institutional support. Additionally, understanding the network structure of the Governance System subsystem will help clarify which farmers are part of unions and how information travels within and between groups. Researchers, practitioners, and stakeholders should also operationalize the SES framework by disentangling the Actor subsystem. For example, understanding how the actors use their current farming technology as opposed to the *Azolla*-rice practice will help determine the available resources and if they are resourced constrained. In general, researchers, practitioners and stakeholders should prioritize working together and combining experiences and perspectives to fill in the missing pieces of *Azolla*-rice farming as a social-ecological system to explore the potential on the biophysical and social side of *Azolla*-rice farming.

## Conclusion

Agriculture is important to Africa’s economy and accounts for the majority of livelihood and wellbeing across the continent [56]. Africa is therefore a “hot spot” for the impacts of climate variability and change due to potentially devastating effects on crop production and food security [57]. Our results provide useful insights to anticipate the presence and productivity of *A*. *pinnata* and *A*. *filiculoides* in Africa for the application of *Azolla*-rice farming as a sustainable agricultural practice under current and future climate change. The use of continental suitability maps can serve as a powerful resource to help local stakeholders establish areas of high and low *Azolla* suitability for regions considering *Azolla* as a biofertilizer for rice cultivation. Further studies should consider collaborating with local stakeholders for bidirectional learning to understand how societies can adopt new agricultural practices based on their goals and priorities. This type of work could be mobilized using the social-ecological systems framework by implementing interview data to understand how attitudes, customs, and social institutions influence *Azolla*-rice uptake. This would get us closer to understanding how to build a more sustainable world by understanding key interactions between humanity and nature. Moreover, this study can be applied to other aquatic species across the globe that are potential biofertilizer candidates.

## Supporting information

S1 Data Recorded article name, *Azolla* species, strain, temperature, initial and final time, relative growth rate, standard deviation, and other environmental conditions tested with temperature used in this study. (XLSX)

S1 Fig Estimated relative growth rate based on mean annual temperatures for Azolla pinnata (A) and A. filiculoides (B) computed from the parameterized thermal performance curves of relative growth rate on a white-green scale. Lower suitability habitats are denoted by yellow while highly suitable habitats are denoted by dark green. Most regions in Africa can support the growth of both Azolla spp. We predicted that the Sahel region (denoted in the black box) will decrease in habitat suitability for Azolla spp. because Azolla growth declines at high temperatures and this is the hottest region in Africa.
(TIF)

S2 Fig *Azolla* spp. suitability shown under two Representative Concentration Pathways (RCPs): RCP 4.5 and RCP 8.5 averaged for year 2050. *A*. *pinnata* is expected to perform better under RCP 4.5 (A) than RCP 8.5 (C) in the Sahel region (denoted in the black box). *A*. *filiculoides* is also expected to have greater suitability under RCP 4.5 (B) than RCP 8.5 (D). Areas in dark green describe more suitable habitats for *Azolla* spp.
(TIF)
