# Supplementary figures and images for: Mapping current and future habitat suitability of Azolla spp., a biofertilizer for small-scale rice farming in Africa
Source: PLoS One. 2023 Dec 18;18(12):e0291009. doi: 10.1371/journal.pone.0291009 (PMC10727437; doi:10.1371/journal.pone.0291009)

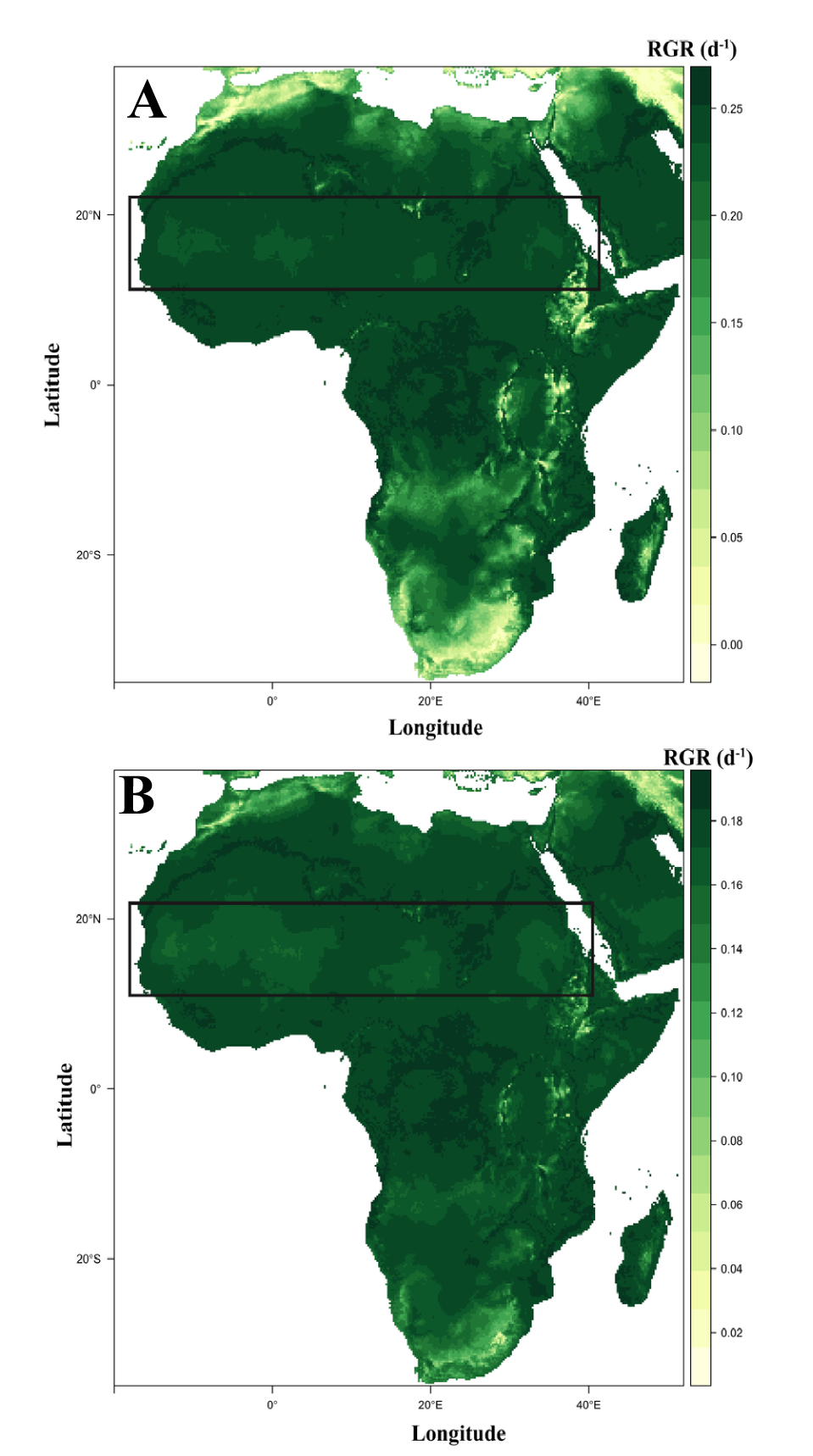

Supplement: S1 Fig — Estimated relative growth rate based on mean annual temperatures for Azolla pinnata (A) and A. filiculoides (B) computed from the parameterized thermal performance curves of relative growth rate on a white-green scale. Lower suitability habitats are denoted by yellow while highly suitable habitats are denoted by dark green. Most regions in Africa can support the growth of both Azolla spp. We predicted that the Sahel region (denoted in the black box) will decrease in habitat suitability for Azolla spp. because Azolla growth declines at high temperatures and this is the hottest region in Africa. (TIF) [file pone.0291009.s002.tif]

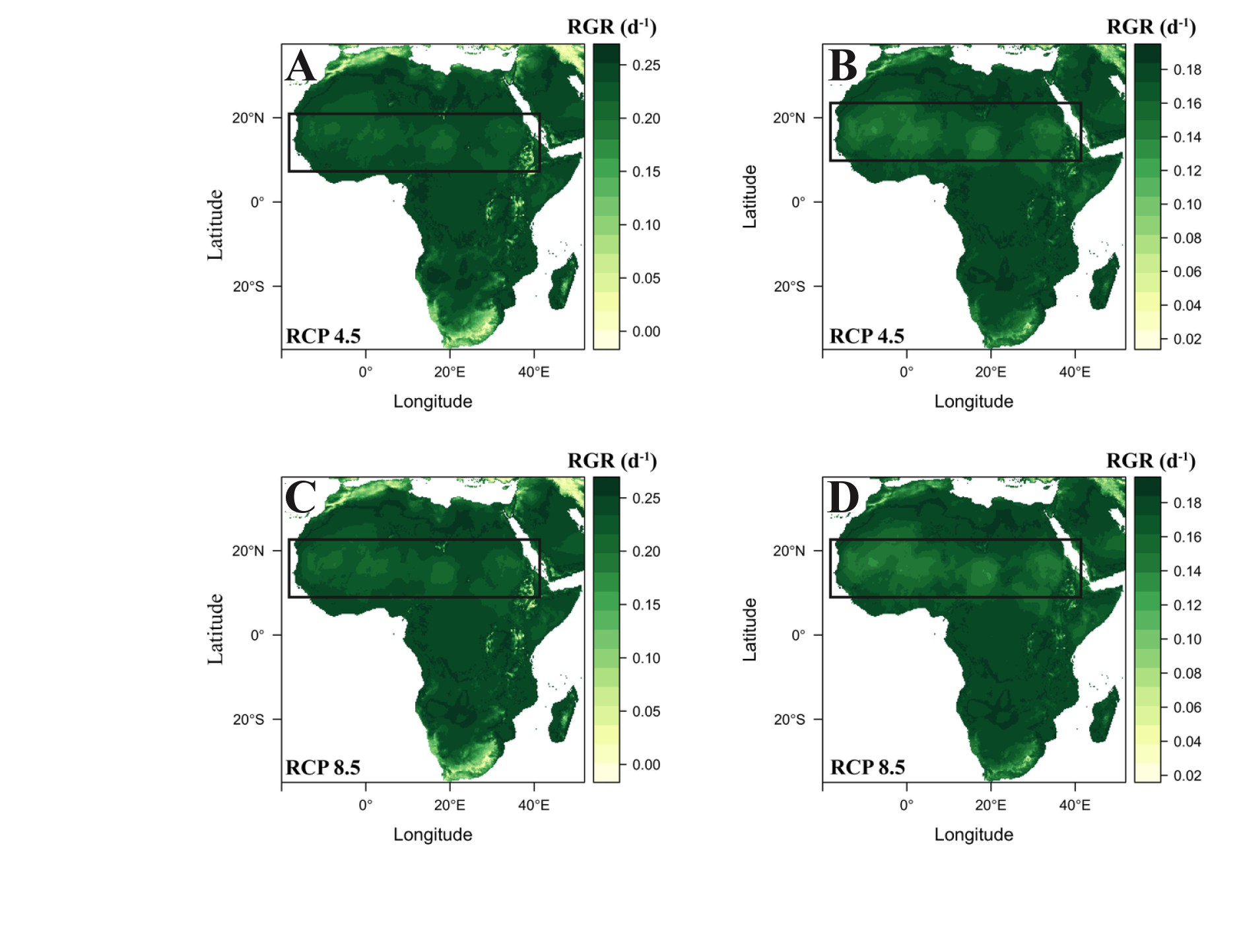

Supplement: S2 Fig — A. pinnata is expected to perform better under RCP 4.5 (A) than RCP 8.5 (C) in the Sahel region (denoted in the black box). A. filiculoides is also expected to have greater suitability under RCP 4.5 (B) than RCP 8.5 (D). Areas in dark green describe more suitable habitats for Azolla spp. (TIF) [file pone.0291009.s003.tif]
